# A Study on Cytotoxic and Apoptotic Potential of a Triterpenoid Saponin (3-O-*α*-L-Arabinosyl Oleanolic Acid) Isolated from* Schumacheria castaneifolia* Vahl in Human Non-Small-Cell Lung Cancer (NCI-H292) Cells

**DOI:** 10.1155/2017/9854083

**Published:** 2017-11-13

**Authors:** Sameera R. Samarakoon, Meran K. Ediriweera, Chukwumaobim Daniel Uzochukwuwulu Nwokwu, Chamara Janaka Bandara, Kamani H. Tennekoon, Poorna Piyathilaka, D. Nedra Karunaratne, Veranja Karunaratne

**Affiliations:** ^1^Institute of Biochemistry, Molecular Biology and Biotechnology, University of Colombo, 90 Cumaratunga Munidasa Mawatha, 03 Colombo, Sri Lanka; ^2^Department of Chemistry, Faculty of Science, University of Peradeniya, Peradeniya, Sri Lanka; ^3^Department of Biotechnology, Faculty of Agriculture and Plantation Management, Wayamba University of Sri Lanka, Makadura, Gonawila, Sri Lanka; ^4^Sri Lanka Institute of Nanotechnology, Mahenwatta, Pitipana, Homagama, Sri Lanka

## Abstract

Lung cancer is the major cause of cancer death among men. A number of natural compounds have proven to be useful in the treatmet of lung cancer. This study was aimed to determine cytotoxic and apoptotoic effects of a natural compound 3-O-*α*-L-arabinosyl oleanolic acid (3-O-L-AO) isolated from* Schumacheria castaneifolia* in non-small-cell lung cancer (NCI-H292) cells. Cytotoxic effects of 3-O-L-AO were determined by Sulforhodamine B (SRB) assay and apoptotic effects were tested by evaluating (a) apoptotsis related morphological changes, (b) caspase 3/7 activity, and (c) expression of* Bax, p53, and survivin* genes. Oxidative stress markers (reactive oxygen species (ROS), glutathione-S-transferase (GST), and glutathione (GSH)) were also analysed in 3-O-L-AO treated NCI-H292 cells. 3-O-L-AO exerted potent cytotoxic effects in NCI-H292 cells while being less cytotoxic to normal lung (MRC-5) cells. Exposure to 3-O-L-AO caused upregulation of* Bax* and* p53* and downregulation of* survivin* in NCI-H292 cells. Activation of caspase 3/7 and morphological features related to apoptosis further confirmed 3-O-L-AO induced apoptosis. Furthermore, elevated ROS and GST levels and decreased GSH levels suggested 3-O-L-AO can induce apoptosis, possibly causing oxidative stress in NCI-H292 cells. Overall results suggest that 3-O-L-AO can be considered as an effective anticancer agent for the treatment of lung cancer.

## 1. Introduction

Lung cancer ranks as the second most commonly diagnosed cancer and major cause of cancer death among men worldwide [1, 2]. Non-small-cell lung cancer (NSCLC) and small-cell lung cancer (SCLC) are the two major types of lung cancer [3]. Non-small-cell lung cancer is the most common form of lung cancer accounting for 85 to 90% of cases [3]. Smoking, genetic factors, some toxic gases, heavy metals, air pollution, and radon gas are implicated as main causes for lung cancer [2]. Radiotherapy, chemotherapy, immunotherapy, and surgery are the commonly used treatment options for lung cancer. Although chemotherapy is the most commonly used treatment, several drawbacks are associated with chemotherapeutic agents such as toxicity, limited efficacy, and drug resistance [2]. Therefore, it is essential to search for new treatment options for lung cancer with fewer side effects. Plants have been recognized as the main source of drugs for many diseases including cancer since ancient times and plant based remedies are known to cause less side effects [4]. Many plant crude extracts have been identified as cytotoxic to cancer cells. However, identification of compounds from such crude extracts with anticancer effects and elucidation of their molecular pathway(s) of action are mandatory for future development of these extracts as cancer therapeutics. Currently used anticancer drugs such as taxol, camptothecin, epipodophyllotoxin, and vinblastine are derived from plant sources and there are several more plant derived anticancer compounds in clinical trials [5].

**Figure 1:**
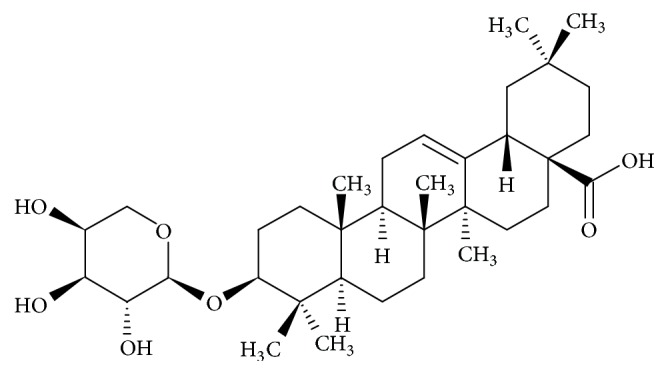
Chemical structure of 3-O-*α*-L-arabinosyl oleanolic acid (3-O-L-AO).

Sri Lanka is a biodiversity hotspot with 894 endemic plant species, thus providing a rich source to identify novel drug leads [6].* Schumacheria castaneifolia* (family Dilleniaceae) which is mainly found in rain forests is a plant endemic to Sri Lanka [7]. A recent study carried out by Jayarathna et al., 2016 [6], has shown cytotoxic and antioxidant effects of chloroform and ethyl acetate extracts of stem bark of* S. castaneifolia* in estrogen receptor positive (MCF-7) and triple negative breast cancer (MDA-MB-231) cells. Another study conducted by Pamunuwa et al., 2015 [8], has reported antioxidant properties of liposomal nanoparticles prepared from the methanol extract of stem bark of* S. castaneifolia*. Isolation and structure elucidation of of several compounds from the leaves of* S. castaneifolia *have also been reported [9, 10]. 3-O-*α*-L-Arabinosyl oleanolic acid (3-O-L-AO), a triterpenoid saponin, is one of the compounds isolated from leaves of* S. castaneifolia* (Figure 1). Moderate antibacterial effects of 3-O-L-AO against* Staphylococcus aureus* and* Escherichia coli* have been reported [9]. However, there are no investigations on anticancer properties of 3-O-L-AO. Therefore, the present study was designed to evaluate possible* in vitro* anticancer effects of 3-O-L-AO in non-small-cell lung cancer cells (NCI-H292).

## 2. Materials and Methods

### 2.1. Materials

Medium (Dulbecco's Modified Eagle's Medium (DMEM)) required for cell culture was purchased from Invitrogen (Carlsbad, CA, USA). Two cell lines (NCI-H292 (small-cell lung cancer cells) and MRC-5 (normal human lung fibroblast cells)) and fetal bovine serum (FBS) were purchased from American Type Culture Collection (ATCC), Rockville, MD, USA. Caspase-Glo 3/7 assay kit and all the reagents required for cDNA synthesis were purchased from Promega, USA. All other chemicals used in this study were purchased from Sigma Aldrich Chemical (St. Louis, MO, USA) unless otherwise indicated. 3-O-L-AO isolated in a previous study carried out by Bandara et al. 2015 was used for the present study [9].

### 2.2. Cell Culture

Two cell lines were cultured in DMEM supplemented with 10% FBS and 0.1% antibiotics in T-25 flasks. Flasks were incubated in a humidified atmosphere of 5% CO_2_ at 37°C.

### 2.3. Cytotoxicity Assays

Sulphorhodamine B (SRB) assay [11, 12] was used to dertmine cytotoxic effects of the compound (3-O-L-AO) in NCI-H292 and MRC-5 cells. Cells (5 × 10^3^/well) were seeded in 96-well tissue culture plates and incubated for 24 h. After 24 h incubation, cells were treated with 3-O-L-AO (0.78–25 *μ*g/mL) and incubated for 24, 48, and 72 h. Paclitaxel (0.47–15 *μ*g/mL) was used as the positive control. Cells were washed with PBS for three times and then fixed by adding 50% trichloroacetic acid (TCA) to each well followed by washing five times with tap water. After fixing, cells were stained with 0.4% SRB dye for 20 min followed by washing five times with 1% acetic acid. Tris-base (10 mM) was then added to each well and plates were kept on a plate shaker for 1 h at room temperature. The absorbance was measured using a microplate reader (Synergy HT, USA) at wavelength of 540 nm.

### 2.4. Morphological Changes

Morphological changes of lung cancer cells (NCI-H292) and normal fibroblasts (MRC-5) cells after exposure to 3-O-L-AO were captured using inverted phase-contrast microscope (Olympus CKX41, Japan).

### 2.5. Morphological Changes Related to Apoptosis Observed under Fluorescence Microscopy

NCI-H292 lung cancer cells (2 × 10^4^ cells/well) cultured on coverslips were treated with 3-O-L-AO (2.5, 5, and 10 *μ*g/mL) for 24 h. After incubation, cells were washed with phosphate buffer saline (PBS) and fixed with 4% formaldehyde for 15 min. Fixed cells were again washed with PBS and stained with acridine orange/ethidium bromide as previously described by us [11]. Stained cells were observed under a fluorescence microscope (Olympus, BX51TRF, Japan).

### 2.6. Caspase 3/7 Activity Assay

Caspase 3/7 activity in NCI-H292 cells was measured using the Caspase-Glo 3/7 assay kit according to manufacturer's protocol [11]. Prior to the assay, NCI-H292 cells were treated with three doses of 3-O-L-AO (0.5, 1, and 2 *μ*g/mL) and incubated for 24 h.

### 2.7. Oxidative Stress Markers

Evaluation of oxidative stress markers (reactive oxygen species (ROS), glutathione-S-transferase (GST), and glutathione (GSH)) after exposure to 3-O-L-AO in NCI-H292 cells was determined as previously described by us [11].

### 2.8. Reactive Oxygen Species (ROS)

NCI-H292 cells (20,000 cells/well) were plated in 96-well cell plates and incubated for 24 h. Following incubation, cells were treated with 3-O-L-AO (0.5, 1, and 2 *μ*g/mL) and incubated for 24 h. Nitro Blue Tetrazolium (NBT) solution (1 mg/mL, 20 *μ*L) was then added to each well and incubated for 1 h at 37°C. Blue formazan formed was then solubalized after adding 100 *μ*L of dimethyl sulfoxide (DMSO) and the absorbance was read at 620 nm.

### 2.9. Glutathione (GSH) Levels

NCI-H292 cells were (1 × 10^5^ cells/well) exposed to 3-O-L-AO (0.5, 1, and 2 *μ*g/mL) for 24 h. After incubation, cells were collected by trypsinization. Collected cell pellets were mixed with ice-cold KCl (2 mL) and 0.5 M EDTA (1 mL) and sonicated for two hours. Cell lysate was then centrifuged at 10,000 g for 15 min to obtain a clear supernatant and it was kept on ice throughout the experiments. Supernatent (75 *μ*L) was mixed with 75 *μ*L of 5% sulfosalicylic acid, 20 *μ*L of 4% DTNB [5,5-dithiobis-(2-nitrobenzoic acid)], and 30 *μ*L of 0.1 M potassium phosphate buffer (pH 8.0) and incubated for 30 min. Absorbance was then recorded at 412 nm and glutathione levels were calculated as nM/mg of cell lysate protein with the help of a GSH standard curve.

### 2.10. Glutathione-S-Transferase Activity (GST)

An aliquot of cell lysates prepared for GSH assays was used to analyse GST activity. Glutathione and CDNB (1-chloro-2,4-dinitrobenzene) were served as substrates in this assay. A mixture was prepared by mixing 490 *μ*L of PBS (pH 6.5), 5 *μ*L of CDNB (100 mM), and 5 *μ*L of GSH (100 mM); 180 *μ*L of this mixture was mixed with 20 *μ*L of cell lysate and incubated for 5 min at 37°C. Absorbance was recoreded at 340 nm for 5 min (1-minute time intervals). Quantity of GST catalyzing the oxidation of 1 millimole of 1-chloro-2,4-dinitrobenzene per minute at 37°C was considerd as one unit of GST activity.

### 2.11. Quantitative Real-Time PCR

NCI-H292 cells (2 × 10^5^ cells/mL) grown in T-25 tissue culture flasks were treated with 3-O-L-AO (1 and 2 *μ*g/mL) and incubated for another 24 h. TRIzol reagent was used to extract RNA as described by Ediriweera et al., 2016 [13]. Extracted RNA (2 *μ*g) was incubated with 50 ng of random primers and 13.5 *μ*L of PCR water for 5 min at 70°C. cDNA synthesis was then carried out after mixing with a maxter mix containing 5 *μ*L MMLV (Moloney Murine Leukemia Virus Reverse Transcriptase) reaction buffer, 5 *μ*L 10 mM dNTP mix, 25 units of RNasin, and 200 units of MMLV RT enzyme, following incubation at 37°C for 60 min, in a total volume of 25 *μ*L. Stratagene Mx3000P real-time PCR system (Agilent Technologies, CA, USA) was used to carry out real-time PCR. Primers used have been previously described [13]. For real-time PCR, 2 *μ*L of cDNA was mixed with Master Mix containing 0.5 *μ*L of forward and reverses primers, 12.5 *μ*L SYBR Green Master Mix (Eurogentec, Seraing, Liège, Belgium), and 9.5 *μ*L PCR water. The thermal cycle programme set for real-time PCR include stage 1, 95°C for 10 min and stage two consisting of three steps: denaturation at 95°C for 30 s, annealing at 56°C for 1 min, and extension at 72°C for 30 s and stage 2 was repeated for 40 cycles. GAPDH (glyceraldehyde 3-phosphate dehydrogenase) was used as the internal house keeping gene. Gene expression was quantified using the method described by Livak and Schmittgen, 2001 [14].

### 2.12. Statistical Analysis

GraphPad Prism 6.0.1 software (GraphPad Software Inc., San Diego, CA, USA) was used for statistical analysis. The results have been expressed as mean ± SD of three individual experiments. One-way analysis of variance (ANOVA) with Tukey's multiple comparisons test (secondary test) was used to analyse significant differences. *p* < 0.05 was considered significant.

## 3. Results and Discussion

### 3.1. Cytotoxic Potential of 3-O-L-AO in Non-Small-Cell Lung Cancer (NCI-H292) and Normal Lung Fibroblast (MRC-5) Cells

**Figure 2:**
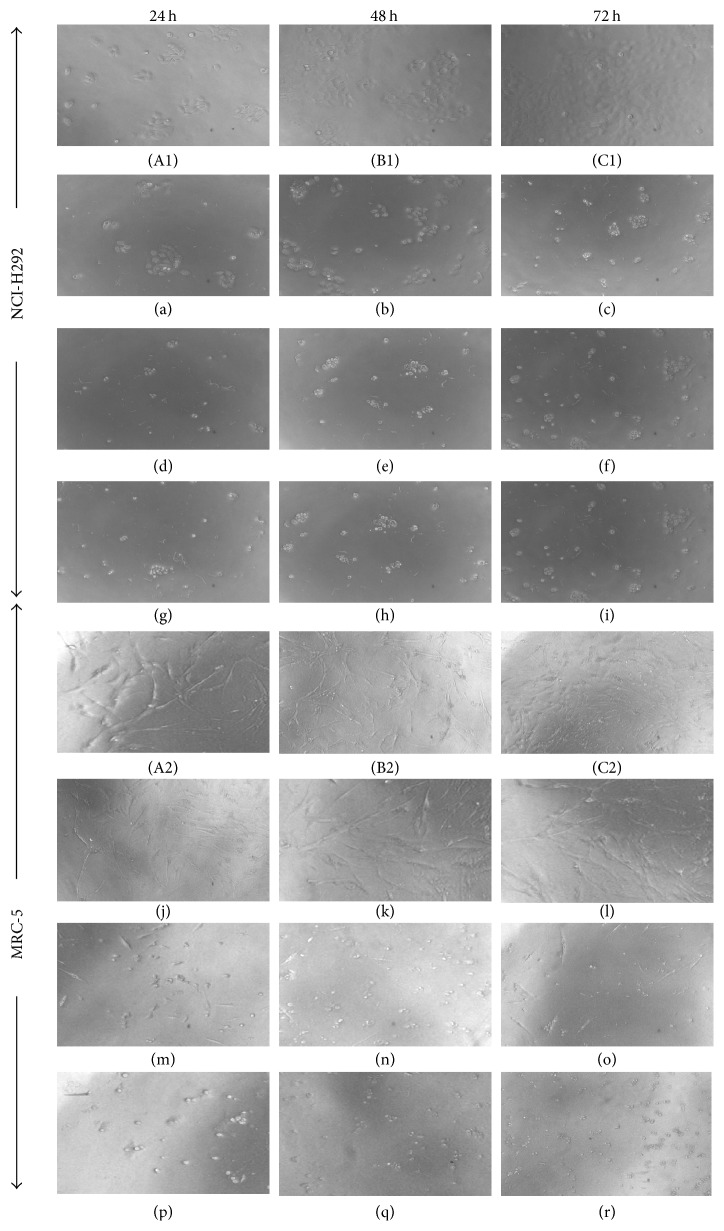
Cytomorphological images of NCI-H292 and MRC-5 cells after exposure to 3-O-L-AO. (A1), (B1), (C1), (A2), (B2), and (C2) are untreated controls. (a), (b), (c), (j), (k), and (l) treated with 1.5 *μ*g/mL; (d), (e), (f), (m), (n), and (o) treated with 6.25 *μ*g/mL; and (g), (h), (i), (p), (q), and (r) treated with 12.5 *μ*g/mL.

According to the results obtained from the SRB assay (Table 1), it is evident that 3-O-L-AO can inhibit lung cancer cell proliferation in a time dependant manner. Cytotoxic effects of 3-O-L-AO in normal lung fibroblast were less compared to lung cancer cells at all three incubation periods (24, 48, and 72 h). Moreover, 3-O-L-AO exhibited a higher cytotoxic effect in lung cancer cells and a lower cytotoxic effect in normal lung fibroblast cells compared to the positive control paclitaxel. Cytomorphological images (Figure 2) following 3-O-L-AO treatment also illustrated a time and dose-dependent inhibition of lung cancer and normal lung fibroblast cells by 3-O-L-AO. Less amount of cells was observed in treated experiments when compared to untreated controls. Considerable morphological changes observed in 3-O-L-AO treated lung cancer and lung fibroblast cells such as decreased cell volume and rounding up cells.

### 3.2. Induction of Apoptosis by 3-O-L-AO in Non-Small-Cell Lung Cancer Cells

As initial cytotoxic assays with 3-O-L-AO confirmed potent cytotoxic effects in non-small-cell lung cancer cells, further studies such as acridine orange (AO) ethidium bromide (EB) staining, estimation of caspase 3 and 7 activities, and evaluation of mRNA expression levels of selected apoptotic related genes cells were carried out to confirm whether exposure of non-small-cell lung cancer cells to 3-O-L-AO can induce apoptosis.

#### 3.2.1. AO/EB Staining

**Figure 3:**
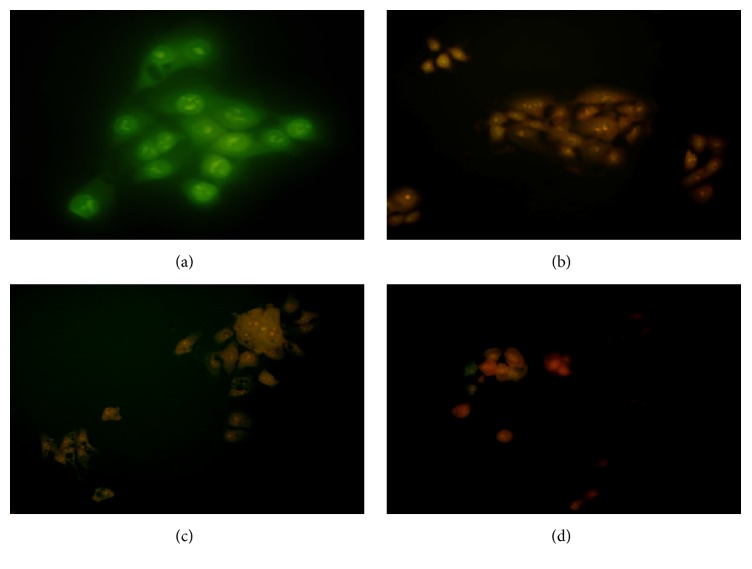
NCI-H292 cells (exposed to 3-O-L-AO) stained with AO/EB and observed under fluorescence microscope showing evidences of apoptosis. (a) Untreated control; (b) treated with 2.5 *μ*g/mL; (c) treated with 5 *μ*g/mL; and (d) treated with 10 *μ*g/mL.

3-O-L-AO treated NCI-H292 cells stained with AO/EB showed morphological changes of apoptosis. Untreated control cells appeared in green as acridine orange (AO) permeates these cells whereas apoptotic cells stain in yellow/red as ethidium bromide (EB) permeates these cells (Figure 3). Results indicated a dose dependent apoptotic induction in 3-O-L-AO treated NCI-H292 cells.

#### 3.2.2. Caspase 3/7 Activity

**Figure 4:**
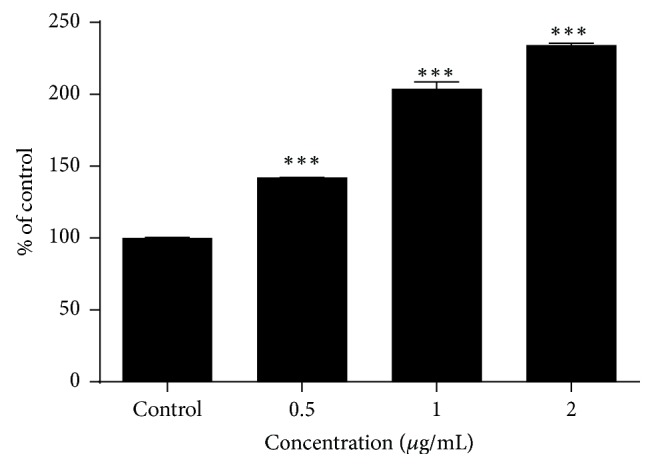
Caspase 3/7 activation in 3-O-L-AO treated NCI-H292 cells (24 h). ^*∗∗∗*^*p* < 0.0001.

Activated caspases 3 and 7 have been recognized as executioner caspases which can initiate apoptosis. Increased caspase 3/7 activity in 3-O-L-AO treated NCI-H292 was observed at 24 h incubation period. Significant (*p* < 0.0001) increase in caspase 3/7 activity was observed at all three doses tested compared to the untreated control (Figure 4).

### 3.3. Effects of 3-O-L-AO on the Expression of Apoptotic Related Genes

**Figure 5:**
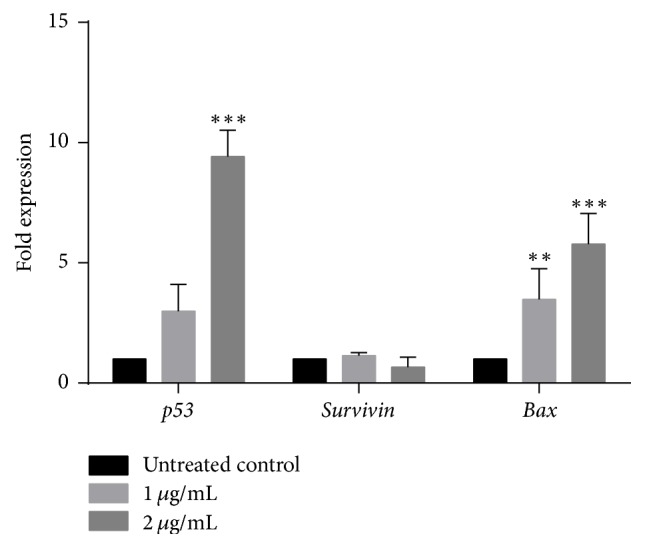
Regulation of apoptosis related genes* (p53, Bax, and survivin)* in NCI-H292 cells after exposure to 3-O-L-AO for 24 h.** (**^*∗∗∗*^*p* < 0.0001; ^**∗∗**^*p* < 0.001**)**.

According to the results obtained in the present study, upregulation of tumor suppressor* p53* gene was observed in 3-O-L-AO treated NCI-H292 cells at both doses tested (1 and 2 *μ*g/mL). However, significant (*p* < 0.05) upregulation was only observed at 2 *μ*g/mL of 3-O-L-AO. Significant (*p* < 0.001 and *p* < 0.0001) upregulation of proapoptotic* Bax* gene was also observed in 3-O-L-AO treated NCI-H292 cells at both doses tested (1 and 2 *μ*g/mL). 3-O-L-AO also caused downregulation of survivin gene which can encode an inhibitor of apoptosis at both the doses tested. However this downregulation was not significant (Figure 5).

### 3.4. Effects of 3-O-L-AO on Oxidative Stress Markers

**Figure 6:**
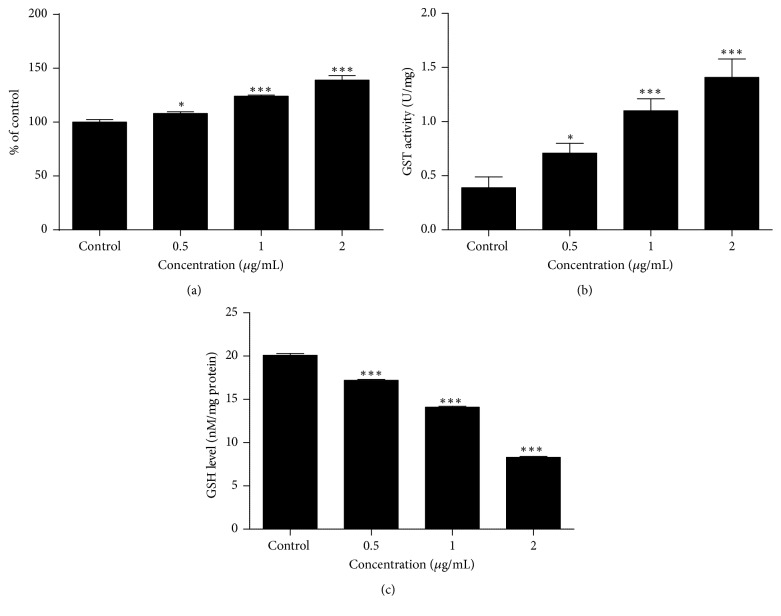
Markers of oxidative stress (ROS, GSH, and GST) after treatment of NCI-H292 cells with 3-O-L-AO for 24 h. (a) ROS levels; (b) GST activity; and (c) GSH levels. ^**∗∗∗**^*p* < 0.0001 and ^**∗**^*p* < 0.05.

As shown in Figure 6 reactive oxygen species (ROS) level was significantly (*p* < 0.05 to *p* < 0.0001) increased at all three doses (0.5, 1, and 2 *μ*g/mL) in 3-O-L-AO treated NCI-H292 cells in a dose dependant manner. ROS are reactive chemical species containing oxygen which can damage live cells and increase apoptosis. Significant reduction in GSH (*p* < 0.0001) and increase in GST (*p* < 0.05 to *p* < 0.0001) levels were also observed at all three doses (3-O-L-AO) in NCI-H292 cells.

## 4. Discussion

A number of triterpenoid saponins with various biological properties have been reported by other authors [15–18]. Among them, cytotoxic and apoptotic potential of natural and synthetic triterpenoid saponins have long been illustrated [19, 20]. Triterpenoid saponins, such as *α*-hederin [21], astragaloside [22], and cucurbitacin [23], are well known examples that have shown promising anticancer effects in several cancer cell lines. However, the present study on the triterpenoid saponin, 3-O-L-AO, is the first study which adressess its cytotoxic and apoptotic potential in lung cancer cells. A compound, with the IC_50_ value of 4 *μ*g/mL or 10 *μ*M, is considerd as a potent anticancer compound [24, 25]. As our compund 3-O-L-AO exhibited IC_50_ values in this margin (in non-small lung cancer cells at 24, 48, and 72 h after incubations), this triterpenoid saponin is suitable to be included in the list of effective anticancer drug leads and, thus, deserves further investigations. Paclitaxel was used only in cell viability studies as the positive control. A positive control was not used for other assays (gene expression, caspase 3/7 assay, and oxidative stress markers) as we mainly focused to elaborate anticancer potential of our selected drug 3-O-L-AO.

Apoptosis (programmed cell death) is the mechanism that removes unwanted cells in biological systems [26]. Morphological changes, shrinkage of cells, generation of apoptotic bodies, condensation of chromatin, and loss of cell organelles are associated with apoptosis [27]. Induction of apoptosis can be triggered via extrinsic and intrinsic pathways [28]. Interaction of specific signaling molecules to death receptors causes initiation of extrinsic pathway, while the intrinsic pathway is initiated by extracellular or intracellular stress. Caspases, a class of protease enzymes, play a prominent role in the regulation of apoptosis. Caspases 2, 8, 9, and 10 are known as initiator caspases while caspaes 3, 6, and 7 are executioner caspases [29]. Evading apoptosis is one of the main features of cancer cells [30]. Therefore, exploration of natural compounds with the ability to induce apoptosis in cancer cells has gained much attention. In the present study apoptotic effects of 3-O-L-AO in NCI-H292 cells were confirmed by observing morphological changes and analysing caspase 3/7 activity. Apart from caspases, several gene products are reported to be involved in apoptosis. Among them, Bcl-2 family of proteins such as Bax (proapoptotic) and Bcl-2 (antiapoptotic) are main proteins in the intrinsic pathway of apoptosis [31]. Activation of initiator caspases is regulated via* Bax*. In apoptosis,* p53* (tumor suppressor gene) is activated through the activation of* Bax*, a Bcl-2 family member [32].* Survivin*, an antiapoptotic protein, is also reported to be regulated by p53 [33]. In the present study, a dose-dependent upregulation of* Bax* and* p53* was observed at both the doses in 3-O-L-AO treated NCI-H292 cells at 24 h incubation. However, upregulation of p53 was only observed at the highest dose. Downregulation of survivin was observed at both the doses tested. However this downregulation was not significant. Induction of apoptosis in cancer cells by generation of reactive oxygen species (ROS) and reduction of endogenous antioxidants has been well discussed [34]. Drugs that generate ROS in cancer cells have been considerd as potential therapeutic strategies to target cancer [35]. Cellular glutathione (GSH) levels are reported to act as protective substances against oxidative stress induced injuries [36]. Therefore, reduction in GSH levels enhances susceptibility to oxidative stress mediated cytotoxicity [37]. GSH conjugate formation with toxic materials in metabolic pathways is catalyzed by glutathione S-transferases (GSTs) [38]. Increase in ROS and GST levels and concomitant decrease in cellular GSH levels imply possible activation of an oxidative stress mediated apoptotic pathway in 3-O-L-AO treated NCI-H292 cells.

## 5. Conclusion

According to the results obtained in the present study, 3-O-L-AO appears to mediate cytotoxic and apoptotic effects through activation of an oxidative stress mediated mechanism in non-small lung cancer cells (NCI-H292). This study provides a rationale for future investigation of anticancer effects of 3-O-L-AO in other* in vitro* and* in vivo* models.

## Figures and Tables

**Table 1:** IC_50_ values (*μ*g/mL) of 3-O-L-AO in NCI-H292 and MRC-5 cells at 24, 48, and 72 h incubation periods.

Cell type	3-O-L-AO (*μ*g/mL)			Paclitaxel (*μ*g/mL)		
	24 h	48 h	72 h	24 h	48 h	72 h
NCI-H-292	4.33	2.69	1.50	8.37	3.28	1.59
MRC-5	5.40	4.59	2.83	1.51	0.96	0.35
